# Identification of QTLs and genes regulating panicle number through genome-wide association studies in *Oryza sativa* L

**DOI:** 10.3389/fpls.2026.1806063

**Published:** 2026-06-09

**Authors:** Fengmei Li, Xiaoqian Ma, Jianyin Xie, Xiaoyang Zhu, Xueqiang Wang, Jianxin Liang, Muhammad Abdul Rehman Rashid, Haifeng Guo, Zhanying Zhang, Jinjie Li, Zichao Li, Jie Luo, Hongliang Zhang

**Affiliations:** 1 School of Tropical Agriculture and Forestry, Hainan University, Haikou, China; 2 College of Agronomy and Biotechnology, China Agricultural University, Beijing, China; 3 Hainan Yazhou Bay Seed Laboratory, Sanya, China; 4 School of Biological Engineering, Xinxiang University, Xinxiang, China; 5 College of Agriculture, Henan University of Science and Technology, Luoyang, China

**Keywords:** candidate gene, genome-wide association study, *Oryza sativa* L., panicle number, quantitative trait loci

## Abstract

**Background:**

Panicle number is a key determinant of grain yield and plant architecture in rice. Here, we investigated quantitative trait loci (QTLs) and genetic factors associated with panicle number using genome-wide association studies.

**Methods:**

We used 3.3 million high-quality single nucleotide polymorphisms and phenotypic data from the Micro Core Collection and Expanded Micro Core Collection, evaluated under two photoperiods across three agroecological zones in China (12 datasets).

**Results:**

We identified 138 QTLs associated with panicle number. Several previously cloned genes, including *OsJAG*, *OsTB1*, *OsAP2-39*, and *D53*, were located within these intervals. QTL effect analysis showed that panicle number increased with favorable allele accumulation and decreased with unfavorable alleles, consistent with quantitative genetic principles. Allelic distribution analysis in landraces and improved varieties revealed a higher frequency of loss alleles than gain alleles. Environmentally stable QTLs with small effects were more suitable for breeding than environmentally sensitive QTL or environmentally stabilizing QTL with larger effects (over three spikes). An integrated approach, including haplotype analysis, pathway analysis, Ho-index unified linkage and association mapping, expression profiling, and T-DNA mutant validation, was used to identify candidate genes within key QTLs. Two cloned genes, *OsARF25* (*qPN12-1*) and *ETR3* (*qPN2-2*), were preliminarily identified as regulators of panicle number. In *qPN7-2*, a gibberellin receptor (*GR*) was identified within the fine-mapped region. Initial functional analysis indicated that *GR* regulates multiple agronomic traits, including panicle number, plant height, panicle length, and secondary branch number. Its expression pattern in Nipponbare roots (RiceXPro database) was similar to that of *D10*, a well-characterized regulator of tiller number.

**Conclusion:**

The identified QTLs and candidate genes provide valuable resources for regulating panicle number in rice through marker-assisted selection, supporting future rice breeding efforts.

## 1 Introduction

Rice (*Oryza sat*iva L.) is the staple food for over half the global population, and developing high-yielding varieties is essential for maintaining food security. Among the three major yield components, panicle number is the most dynamic and fundamental; both excessive and insufficient panicle numbers negatively affect the overall yield (Song et al., 2022; Wang et al., 2024). Panicle number is determined by tiller number during the vegetative phase and panicle percentage during the reproductive stage (Ma et al., 2020). Gene expression in tiller node buds at the root–stem junction regulates tiller number (Shao et al., 2019), while expression in the growing tips of young spikelets influences panicle setting rate, together determining final panicle number (Yan et al., 1998). Low heritability of tiller number and panicle percentage results in overall low heritability of panicle number. External factors, including temperature, light, planting density, and nitrogen, and internal factors, such as hormones and nutrient allocation, jointly affect panicle number (Xing and Zhang, 2010; Wang et al., 2017). These combined effects lead to substantial phenotypic variation in panicle number within the same variety group, across environments, and between growing seasons (Yang T. et al., 2025).

Significant progress has been made in understanding panicle number through advances in genomics and molecular technologies (Wang and Li, 2019). To date, more than 100 quantitative trait loci (QTLs) have been mapped, and numerous genes regulating panicle or tiller number have been cloned. Most of these genes function through plant hormone pathways, including strigolactones (SLs), gibberellin, auxin, cytokinin, brassinosteroids, and ethylene (Zhang et al., 2023). These hormones interact to form a complex regulatory network that coordinates panicle and tiller development. *MOC1* is linked to multiple phytohormones, such as gibberellin and abscisic acid, and is essential for the formation of leaf axillary meristems during both vegetative and reproductive stages (Li et al., 2003). Interactions between *MOC1* and other cloned genes have been characterized (Zuo and Li, 2014), including *MIP1* (Sun et al., 2010), *TAD1*/*TE* (Xu et al., 2012), *MOC3* (Lu et al., 2015), *FON1* (Shao et al., 2019), *OsTB1* (Guo et al., 2013), *OsMADS57*, *D14*, and *LAX2* (Tabuchi et al., 2011). SLs are a class of terpenoid-derived plant hormones that are essential for the initiation and elongation of tiller buds. They are synthesized predominantly in the roots and transported to aerial tissues (Wang F. et al., 2020). Carotene is the primary substrate for the biosynthesis of SLs, 5-deoxystrigol, and related compounds, and this biosynthetic pathway is regulated by *HTD1*, *D27* (Lin et al., 2009), *D10* (Arite et al., 2007), *D17* (Huang et al., 2025), *OsCCD7* (Wang Y. et al., 2020), and *cytochrome P450 monooxygenases*. SL signal transduction follows a “de-inhibition activation” mechanism. Phosphorylation of D14 by DSK1 promotes the assembly of a complex comprising the SL receptor D14 (Guo et al., 2013), the F-box protein D3, and the transcriptional repressor D53 (Hu et al., 2024). This complex mediates SL-triggered ubiquitination and degradation of D53, thereby activating downstream pathways that regulate tiller number (Jiang et al., 2013; Zhou et al., 2013). *IPA1* plays a key role in feedback regulation of *D53* expression following SL induction (Song et al., 2017). Deletion of binding site within *IPA1* for the transcription factor *An-1* mitigates the trade-off between panicle number and size imposed by gene pleiotropy and linkage drag, resulting in increased yield (Song et al., 2022). Approximately two-thirds of cloned genes show mutation-based variation, providing insight into the molecular mechanisms underlying panicle number; however, their application in breeding is limited by low natural polymorphism (Lei et al., 2018; Liu et al., 2020). Therefore, identifying QTLs or genes associated with panicle number in natural populations and elucidating their genetic mechanisms remains essential for improving rice yield.

Accurate phenotypic assessment remains challenging because panicle number is controlled by many minor-effect genes and is strongly influenced by genotype–environment interactions (Ma et al., 2020; Takai et al., 2024). Advances in high-throughput sequencing and bioinformatics have enabled genome-wide association studies (GWAS), which help overcome limitations in mapping loci or candidate genes for environmentally sensitive traits (Yano et al., 2016; Guo et al., 2024; Sun et al., 2022). GWAS offers several advantages, including comprehensive data acquisition, enhanced result precision, and streamlined computational analysis (Alamin et al., 2022). For environmentally sensitive traits, the GWAS phenotype is typically the mean value across multiple individuals per natural variety. This approach improves the efficiency and accuracy of functional gene identification, particularly in the presence of structural variation (Li et al., 2024). Therefore, this study aims to identify various QTLs associated with panicle number using GWAS and to discover novel genes with variation in natural populations. The results will provide insights and genetic resources to help optimize panicle number and improve rice yield potential.

## 2 Materials and methods

### 2.1 Plant materials

GWAS were conducted using 683 natural germplasm accessions, which were divided into two populations: the Micro Core Collection (MCC; 266 geographically and morphologically representative varieties; Zhang et al., 2011) and the Expanded Micro Core Collection (EMCC; 655 accessions). The overlapping germplasm between MCC and EMCC was counted only once in the total count. The EMCC included 238 MCC accessions, 159 parents from global molecular breeding programs, 236 elite Chinese varieties, and 22 additional germplasm accessions. It comprised 396 *indica* and 259 *japonica* accessions; 173 landraces (LAN), 361 improved varieties (IMP), and 121 uncertain varieties; and 398 accessions from China, 235 from 40 other countries, and 22 of uncertain geographic origin. This diverse population exhibits a complex genetic structure, providing a strong basis for identifying elite genes and pyramiding favorable alleles to improve yield (Wang et al., 2022).

### 2.2 Phenotyping

A randomized complete block design with two replicates was used to evaluate panicle number in the EMCC population at two locations in China—Sanya (SY_EMCC_) and Beijing (BJ_EMCC_)—in 2014. Standardized field management guaranteed accurate phenotypic evaluation: healthy seedlings were transplanted at 20 × 25 cm spacing to reduce environmental and genotype interaction effects; a shallow water layer was maintained during tillering to support bud growth; irrigation, fertilization, and pest control followed standard agronomic practices. Panicle number of EMCC was measured at maturity as the mean of five randomly selected plants from the middle row of each accession. Panicles with fewer than 50 filled grains were excluded from the count to ensure phenotypic accuracy. Phenotypic data for the MCC population from Sanya (SY_MCC_) and Changsha (CS_MCC_) in 2013 were obtained from a previous study (Li et al., 2018). Data were analyzed using WPS Office 2025 and IBM SPSS Statistics 19.0 to generate descriptive statistics, including mean, minimum, maximum, standard deviation, coefficient of variation, and Pearson correlation coefficient.

### 2.3 Genome sequencing and single-nucleotide polymorphism identification

Whole-genome sequences for all materials were derived from the 3,000 Rice Genomes Project (Li, 2014; Alexandrov et al., 2015) (The 3,000 rice genomes project, 2014;. Single nucleotide polymorphisms (SNPs) and allele data are available from the Rice SNP-Seek Database (http://www.oryzasnp.org/iric-portal/). Sequencing libraries were constructed using the Illumina Genomic DNA Prep Kit according to the manufacturer’s protocol and sequenced on an Illumina HiSeq 2000 platform at BGI-Shenzhen. The 683 genomes were sequenced at an average sequencing depth of 14×, generating 3.87 TB of high-quality data. Clean reads were aligned to the Nipponbare reference genome (Os-Nipponbare-Reference-IRGSP-1.0) using BWA (Vaillancourt et al., 2013), yielding an average genome coverage and mapping rate were 94.0% and 92.5%, respectively. Approximately 10.7 million SNPs were initially identified using the Genome Analysis Toolkit GATK (UnifiedGenotyper, v2.0-35) and Picard (v1.71). After quality control, 3.3 million high-quality SNPs (minor allele frequency ≥5%, missing rate ≤50% in MCC and EMCC) were retained, with an average density of 8.86 SNPs per kilobase.

### 2.4 GWAS and QTL detection

GWAS for panicle number were conducted on the full population and on the *indica* and *japonica* subpopulations using a compressed mixed linear model to minimize subpopulation structure effects (Zhang et al., 2010). Principal component analysis (PCA) and kinship analysis of 655 varieties were performed in GAPIT using the 3.3 million high-quality SNPs (Lipka et al., 2012). Threshold determination, significant SNP identification, and QTL detection followed Li et al. (2018).

### 2.5 QTL effect analysis

QTL effect was defined as the difference between the phenotype of each allele and the average phenotype of the two alleles at the same locus (Xie et al., 2022). After excluding missing and null phenotypes, QTL effects were calculated using a representative tag SNP selected from significant SNPs within each QTL region using Haploview.jar. QTLs were classified as positive or negative based on the direction of the phenotypic effect.

### 2.6 Candidate gene identification

Factors such as population structure, slow linkage disequilibrium decay, rare alleles, and redundancy or inhibition among SNPs have limited the precision of genetic studies. For example, some functional genes lack significant SNPs, whereas a single GWAS QTL interval may contain dozens to thousands of significant SNPs and candidate genes. To fine-map consistent QTLs between GWAS-associated loci and “hot QTLs” from linkage mapping, we conducted a comprehensive analysis that included haplotype-based variance analysis, pathway analysis, and the ho-index unified linkage and association mapping (Ho-LAMap) (Yu et al., 2017).

“hot QTLs” were identified by dividing the rice genome into 1 Mb bins and calculating the number of mappings per bin based on QTLs for panicle number from previously reported linkage analyses. QTLs detected two or more times were defined as “hot QTLs”. The inclusion of more linkage-derived QTLs improves statistical accuracy; therefore, low-frequency QTLs should not be excluded.

Haplotype-based variance analysis of candidate genes within target QTLs reduces redundancy and interaction effects among multiple functional SNPs within a gene (Yano et al., 2016). The analysis included the following steps: (1) SNP calling for each gene, including the gene body and a 2.5 kb upstream promoter region; if the intergenic distance between adjacent genes was <2.5 kb, the intervening sequence was defined as the promoter region. (2) Filtering SNPs with minor allele frequency ≤5% and missing rate ≥50%. (3) Treating heterozygous genotypes [GT (K), AC (M), AG (R), AT (W), CG (S), CT (Y), NN (N)] as missing and imputing them using the linkage disequilibrium KNNi in TASSEL 5.0; QTL regions were extended by 0.5 Mb on both sides to improve imputation at gene boundaries. (4) Removing intronic SNPs and synonymous coding SNPs. (5) Selecting tag SNPs for each gene using Haploview. (6) Performing ANOVA on haplotypes defined by one or more tag SNPs, separately for *indica* and *japonica* subpopulations, which account for ~95% of population structure; only haplotypes represented by ≥3 accessions were analyzed.

Candidate gene pathway analysis used a proprietary database comprising 1,843 pathways and 13,687 genes, including data from the Kyoto Encyclopedia of Genes and Genomes, developed in collaboration with BGI-Shenzhen through genome sequence alignment.

### 2.7 T-DNA mutant validation of candidate genes

T-DNA mutants of *etr3* and *gr*, along with the wild-type (WT) control, were obtained from the Salk Institute Genomic Analysis Laboratory; additional details are available online (http://www.signal.salk.edu). Phenotypic comparisons between mutants and WT plants were performed with ten independent biological replicates after propagating the lines for two or three generations to obtain sufficient homozygous individuals. Statistical significance was determined using Student’s t-test.

### 2.8 Expression patterns analysis

Candidate gene expression across developmental stages was analyzed using RiceXPro (http://www.ricexpro.dna.affrc.go.jp/).

### 2.9 Statistical analysis

The regression coefficient quantifies the effect of an independent variable on the dependent variable; here, it represents the degree of decrease in the IMP/LAN allele ratio relative to an increase in allele effects.

The determination coefficient reflects the accuracy of the regression.

The effective regression coefficient, defined as the product of the regression coefficient and the determination coefficient, indicates the effective degree of decrease in the IMP/LAN ratio relative to an increase in allele effects.

## 3 Results

### 3.1 Phenotypic variations of MCC and EMCC

**Figure 1:**
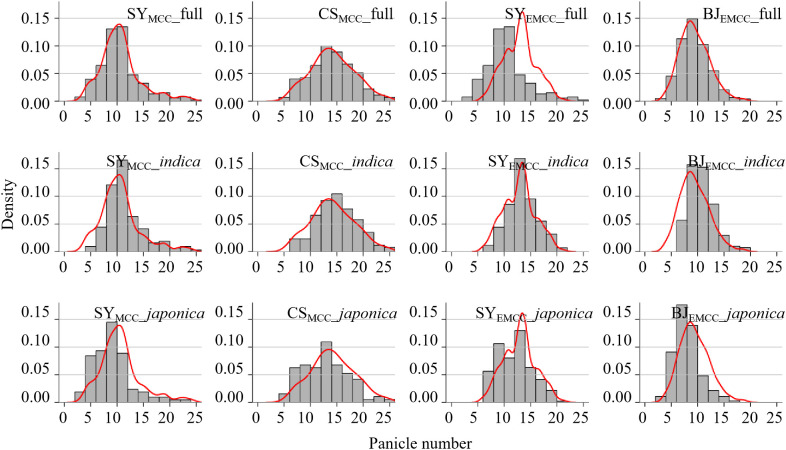
Phenotypic variation in panicle number of the Micro Core Collection (MCC) and Expanded Micro Core Collection (EMCC) across different environments and populations.

Panicle number (panicles with ≥ 50 filled grains) was evaluated at the maturity stage in SY_MCC_, CS_MCC_, SY_EMCC_, and BJ_EMCC_ by manually counting all panicles per plant, with five plants assessed per accession. The trait showed substantial genetic variation and followed a normal distribution (**Figure 1**), with ranges of 3–24.3, 5.3–25, 4.6–21.5, and 3.6–19, respectively (**Supplementary Table 1**). Among the tested varieties, 238 were shared between MCC (266 varieties) and EMCC (655 varieties). Phenotypic correlations across the four environments were analyzed using IBM SPSS Statistics 19.0, and significance was assessed using Student’s t-test. Correlation coefficients (r) among SY_MCC_, SY_EMCC_, and BJ_EMCC_ exceeded 0.6, whereas those between CS_MCC_ and the other environments were approximately 0.4 (**Supplementary Table 2**). These results indicate that panicle number is highly sensitive to environmental variation.

### 3.2 Population structure

PCA showed that PC1–PC10 explained ~90% of the total variation in EMCC population structure, primarily reflecting the indica–japonica divergence. The 655 accessions were clearly separated into two subpopulations: *Oryza sativa* ssp. *indica* (396 varieties) and ssp. *japonica* (259 varieties) (**Supplementary Figure 1**). Kinship analysis of EMCC was consistent with the PCA results (**Supplementary Figure 2**). Detailed information on the MCC population structure has been reported previously (Li et al., 2018).

### 3.3 GWAS results

**Figure 2:**
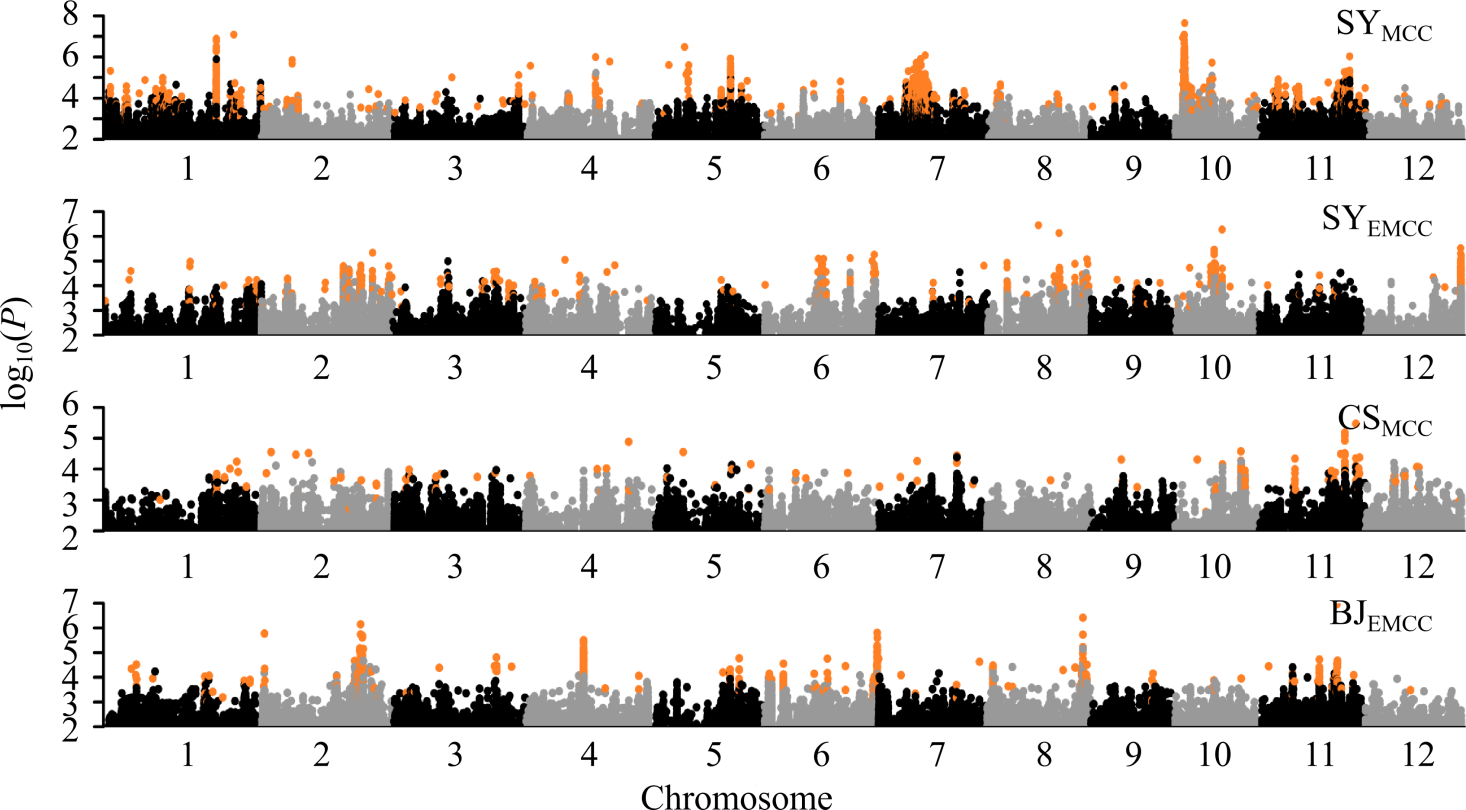
Manhattan plots of GWAS for panicle number in the full populations of SY_MCC_, SY_EMCC_, CS_MCC_, and BJ_EMCC_. Orange dots indicate SNPs with −log_10_(*P*) values exceeding the maximum threshold derived from 1,000 permutations. GWAS: genome-wide association study.

GWAS using a compressed mixed linear model was conducted on the full population and the *indica* and *japonica* subpopulations separately, based on 3.3 million high-quality SNPs and multi-year phenotypic data for panicle number (**Figure 2**; **Supplementary Figures 3**, **4**). In total, 138 QTLs associated with panicle number were identified using permutation-based significance thresholds. Specifically, 33, 17, 14, 10, 8, 13, 34, 28, 15, 20, 25, and 14 QTLs were detected in SY_MCC_, SY_MCC__*ind*., SY_MCC__*jap*., CS_MCC_, CS_MCC__*ind*., CS_MCC__*jap*., SY_EMCC_, SY_EMCC__*ind*., SY_EMCC__*jap*., BJ_EMCC_, BJ_EMCC__*ind*., and BJ_EMCC__*jap*., respectively (**Supplementary Table 3**). The cloned genes *OsJAG*, *OsTB1*, *OsAP2-39*, and *D53* were identified as candidate genes within these QTLs. Significant differences were observed in the QTLs across various populations and environments. The number of QTLs in SY_MCC_, CS_MCC_, SY_EMCC_, and BJ_EMCC_ showed a weak negative correlation with phenotypic mean (*r* = −0.31), suggesting a relatively high proportion of negative-effect QTLs for panicle number. Pairwise comparisons identified 3, 5, 2, 2, 1, and 6 common QTLs between SY_MCC_–CS_MCC_, SY_MCC_–SY_EMCC_, SY_MCC_–BJ_EMCC_, CS_MCC_–SY_EMCC_, CS_MCC_–BJ_EMCC_, and SY_EMCC_–BJ_EMCC_, respectively. The number of shared QTLs was strongly positively correlated (r > 0.77) with inter-population phenotypic correlations, which partly explains that phenotypic similarity among populations is primarily driven by their consistent genetic basis for panicle number.

QTLs consistently detected across multiple populations or environments may play key roles in regulating panicle number. Environmentally sensitive QTLs were defined as those detected only once across environments or years, whereas stable QTLs were detected at least twice. Comparative analysis across multiple GWAS results identified 115 environmentally sensitive QTLs, including 63, 54, 33, and 11 in the full, *indica*, *japonica*, and both subpopulations, respectively (**Supplementary Figure 5**). In contrast, 23 environmentally stable QTLs were identified, including 14, 9, and 7 in the full, *indica*, and *japonica* populations, respectively.

### 3.4 QTL effect analysis

**Figure 3:**
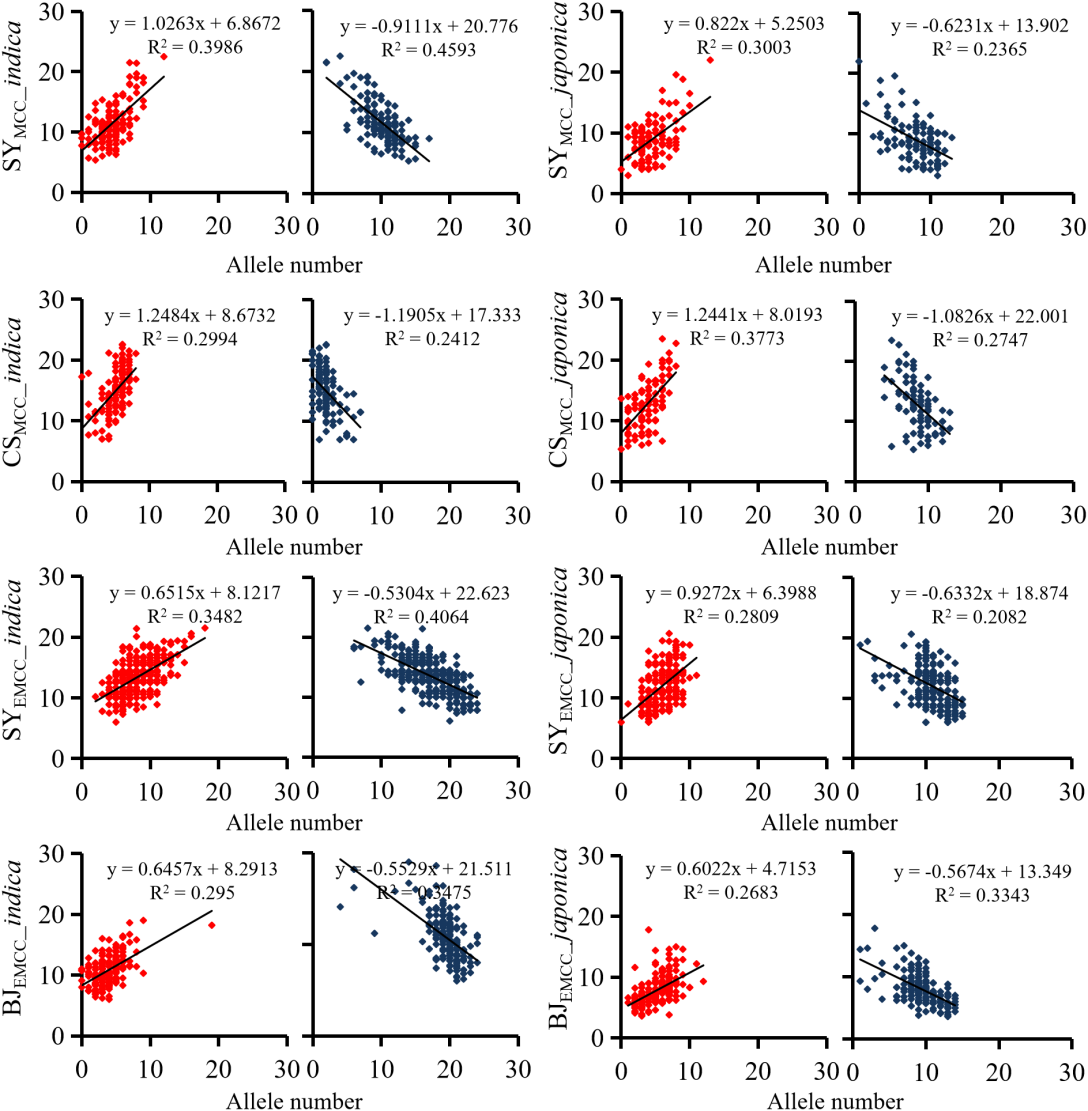
Correlations between allele number and panicle number across different environments and populations. Red and blue dots indicate alleles with positive and negative effects, respectively.

To analyze QTL effects, we quantified the effects of GWAS-identified QTLs across diverse subpopulations and environments, counted the number of alleles with positive or negative effects in each variety, and examined the correlation between phenotypic values and the number of distinct alleles. In all 4 × 4 datasets, panicle number increased with the accumulation of positive-effect alleles and decreased with the accumulation of negative-effect alleles (**Figure 3**), consistent with the principles of quantitative genetics.

**Figure 4:**
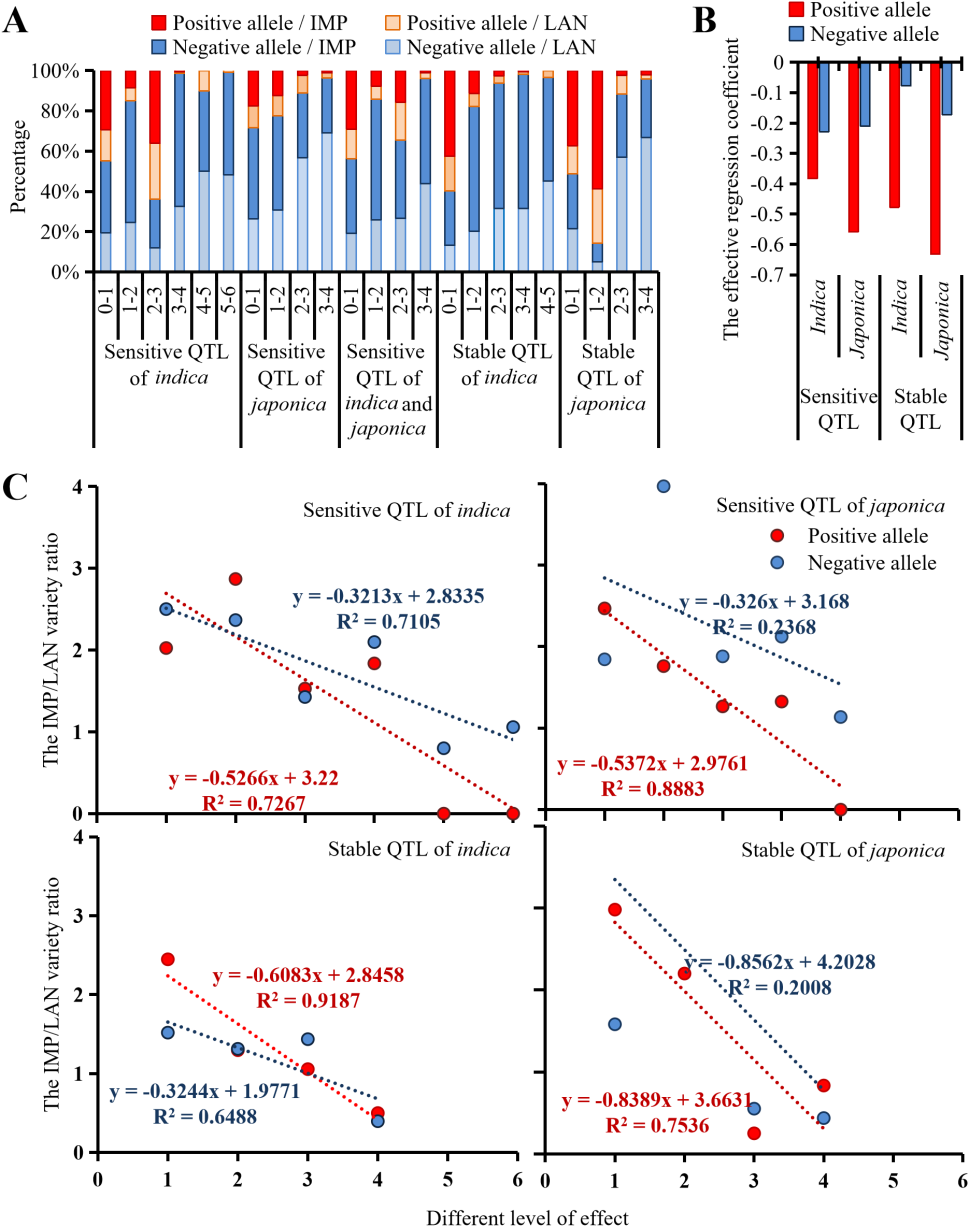
Distribution of alleles with different effect sizes in LAN and IMP (IMP/IML) varieties. **(A)** Percentage of negative and positive alleles in LAN and IMP (IMP/IML) varieties; different ranges indicate different levels of allelic effects. **(B)** Effective regression coefficient between the IMP/LAN variety ratio and the effects of positive or negative alleles of quantitative trait loci (QTLs) detected in different subpopulations. **(C)** Regression of the IMP/LAN variety ratio on the effect of positive or negative alleles in sensitive QTLs of *indica* (upper left), sensitive QTLs of *japonica* (upper right), stable QTLs of *indica* (lower left), and stable QTLs of *japonica* (lower right).

We then classified QTLs into six types: *indica*-sensitive, *japonica*-sensitive, common *indica* and *japonica*-sensitive, *indica*-stable, *japonica*-stable, and common *indica* and *japonica*-stable. QTLs were also grouped by effect size into six intervals: 0–1, 1–2, 2–3, 3–4, 4–5, and 5–6. Based on these groupings, we calculated regression relationships and effective regression coefficients to evaluate the association between the IMP/LAN variety ratio (with positive or negative alleles) and effect levels. Three key patterns emerged (**Figure 4**): (1) Negative-effect alleles were more frequent than positive-effect alleles. (2) Alleles with different effect sizes showed distinct patterns. Positive minor-effect alleles were more common than positive major-effect alleles, whereas the opposite trend was observed for negative-effect alleles. IMP varieties had a higher proportion of minor-effect alleles than LAN varieties, while LAN varieties had more major-effect alleles. These results suggest that minor positive-effect alleles have been widely used in IMP varieties, whereas QTLs with effects >3 panicles, whether stable or environmentally sensitive, remain underutilized in breeding. (3) Stable and sensitive QTLs showed different patterns. Stable QTLs had a higher proportion of positive effects than sensitive QTLs, and the IMP/LAN ratio for stable minor-effect QTLs was higher than for sensitive ones. Collectively, these results indicate that stable minor-effect QTLs are preferentially selected in breeding because they produce consistent effects and support gradual improvement through polygenic trait pyramiding, serving as key genetic loci for developing widely adaptable, high-quality varieties.

### 3.5 Mining of potential candidate genes

The QTLs identified by GWAS were fine-mapped through comprehensive analysis. Among the 56 GWAS QTLs detected at least twice across 12 datasets (**Supplementary Figure 6**) and the 21 “hot QTLs” (**Supplementary Figure 7**; **Supplementary Table 3**), seven overlapped. These loci were located at Chr2:25817675–28086420, Chr2:34522647–35031410, Chr6:29581378–30589079, Chr7:7064297–16101455, Chr7:20146650–20857916, Chr11:9503821–16618570, and Chr12:25833407–26842170. Subsequent analyses focused on these seven QTLs (**Supplementary Table 4**). Haplotype-based variance analysis of associated candidate genes identified 1,170 significant genes (*P* < 0.01).

Pathway analysis of previously cloned genes related to tiller or panicle number provided insight into candidate gene identification within these seven QTLs. Ten cloned genes were involved in six pathways, with the majority involved in “plant hormone signal transduction” and “carotenoid biosynthesis” both known to influence panicle number. Pathway enrichment analysis of all 1,170 genes from the seven QTLs showed that 47 candidate genes from six QTLs were associated with these two pathways (**Supplementary Table 5**). Among these, *qPN2-2*, *qPN7-2*, and *qPN12–1* contained four, seven, and two candidate genes, respectively (**Supplementary Table 6**).

#### 3.5.1 Candidate gene analysis in *qPN2–2* and *qPN12-1*

A detailed analysis of *qPN2-2* (Chr02:34,522,647–35,031,410), which showed strong GWAS signals in CS_MCC__full, CS_MCC__*jap*., and SY_EMCC__full, identified LOC_Os02g56120, LOC_Os02g56140, LOC_Os02g57250 (*OsIAA10*), and LOC_Os02g57530 (*ETR3*) as priority candidate genes (**Supplementary Figures 8A**, **9A**). Based on annotations from the National Center for Biotechnology Information and previous studies, *ETR3* belongs to the same gene family as *ETR2*. Transgenic plants overexpressing *ETR2* show reduced productive tiller percentage and shorter plant height compared with wild type (Wuriyanghan et al., 2009). To explore the biological functions of *ETR3*, a T-DNA insertion mutant was analyzed (**Supplementary Table 7**). The mutant showed a significant reduction in panicle number from 9.15 to 7.8 (14.75%, *P* < 0.05) relative to the wild type (Hwayoung). Additionally, the plant height, panicle length, and secondary branch number of *etr3* significantly decreased by 23.58%, 14.13%, and 33.02%, respectively (*P* < 0.01) (**Supplementary Figures 9C**, **10**). Expression analysis using RiceXPro data further supported a role for *ETR3* in vegetative growth and panicle development. Haplotype analysis showed that *ETR3* variation occurred mainly in *japonica*, where the Hap2 haplotype conferred significantly lower panicle number than other haplotypes (**Supplementary Figure 9B**). The dominant allele distribution in LAN and IMP varieties suggests that the favorable *ETR*3 allele has not been systematically selected in current breeding programs. A complete list of all SNPs utilized for the haplotype analysis is provided in **Supplementary Table 10**, which includes the following information for each SNP: genomic physical position, reference allele, alternative allele, variant type, amino acid substitution, indica frequency (%), and japonica frequency (%).

After integrating *qPN12-1* (Chr12:25,833,407.26,842,170) with significant GWAS signals for SY_EMCC__full and SY_EMCC__*ind.* (**Supplementary Figures 8B**, **11A**), LOC_Os12g41950 (*OsARF25*) and LOC_Os12g42280 (*OsNCED5*) were identified as candidate genes associated with panicle number. A review of the literature showed that the auxin-responsive transcription factor *OsARF25* directly binds the *OsCKX4* promoter, regulates *OsCKX4* (*ren1-D*) expression, and affects tiller number (Gao et al., 2014). In addition, LAX2 disrupts interferes with the OsIAA3–OsARF25 interaction, promoting main stem elongation and inhibiting lateral bud development through the auxin signaling pathway (Zhang et al., 2018). To clarify the biological role of *OsARF25*, T-DNA insertion mutants were analyzed (**Supplementary Table 8**). A mutation in the *OsARF25* exon increased panicle number from 9.15 to 10.78 (17.81%) relative to WT (*P* < 0.01) (**Supplementary Figures 11C**, **12**). Concurrently, the plant height, panicle length, and secondary branch number of *osarf25* were significantly reduced by 4.61%, 6.51%, and 26.40%, respectively (*P* < 0.01). Haplotype analysis indicated genetic variation for panicle number in both *indica* and *japonica*, with clear differences between Hap3 and Hap6 (**Supplementary Figure 11B**). Details of all SNPs used for haplotype analysis are shown in **Supplementary Table 10**. Allele distribution in LAN and IMP varieties showed that the favorable *OsARF25* allele is widely present in IMP varieties and has been extensively used in rice breeding.

#### 3.5.2 *GR* is crucial for regulating panicle number in rice

**Figure 5:**
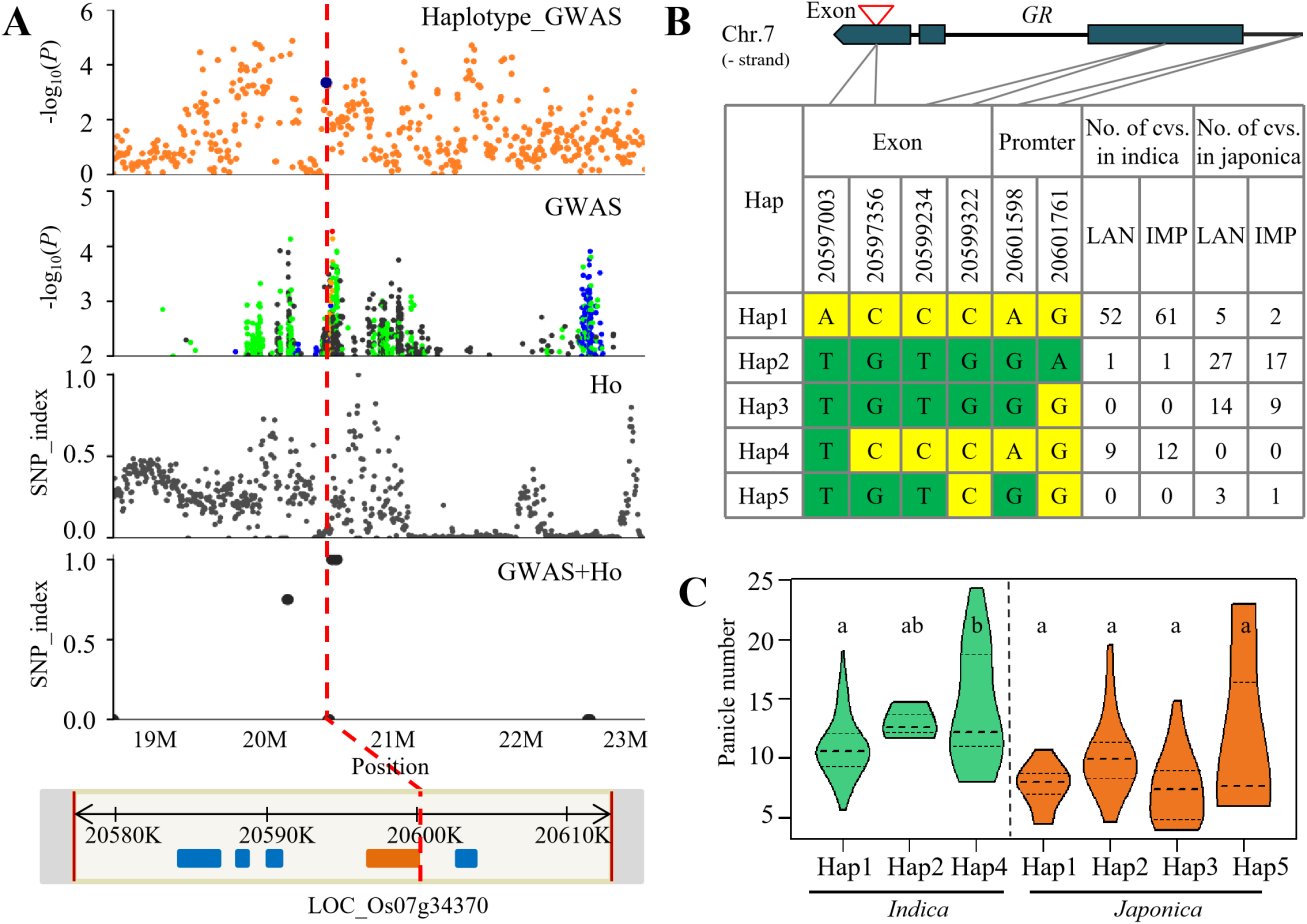
Manhattan plot and haplotype analysis of *GR*. **(A)** Haplotype-based variance analysis of candidate genes within *qPN7-2*, with dark blue dots indicating the *GR* gene; GWAS analysis and linkage disequilibrium (r²) analysis between the lead SNP and all SNPs in the QTL region (red: *r*^2^ > 0.8; orange: 0.8 > *r*² > 0.6; blue: 0.6 > *r*² > 0.4; green: 0.4 > *r*² > 0.2; gray: *r²* < 0.2); Ho-index between the two parents; results of Ho-LAMap. **(B, C)** Gene structure and haplotype analysis of *GR*. Red triangle indicates the T-DNA insertion site.

*qPN7-2* (Chr07:20,146,650.20,857,916) showed the strongest association signal and was consistently detected across multiple GWAS, indicating a robust effect on panicle number. Seven candidate genes were identified within a 40 Kb region based on haplotype variance and pathway analyses. Fine mapping of *qPN7–2* using Ho-LAMap was conducted with polymorphic SNPs between the parental genotypes of Yuefu × IRAT109. LOC_Os07g34370 (GA receptor [*GR*]) was included in the QTL interval (**Figure 5A**). *GR* is involved in plant hormone signal transduction and shows an expression pattern similar to *D10* in RiceXPro (**Supplementary Figure 13**), a known regulator of tiller number (Arite et al., 2007). Haplotype analysis identified five *GR* haplotypes (**Figures 5C, B**). Variation associated with panicle number was detected only in indica, with no haplotype differences in *japonica*. Significant differences in panicle number were observed between Hap1 and Hap4, and the causal functional SNP(s) within the *GR* gene remain to be identified and validated by additional functional assays. The detailed information of all SNPs used for haplotype analysis is summarized in **Supplementary Table 10**.

**Figure 6:**
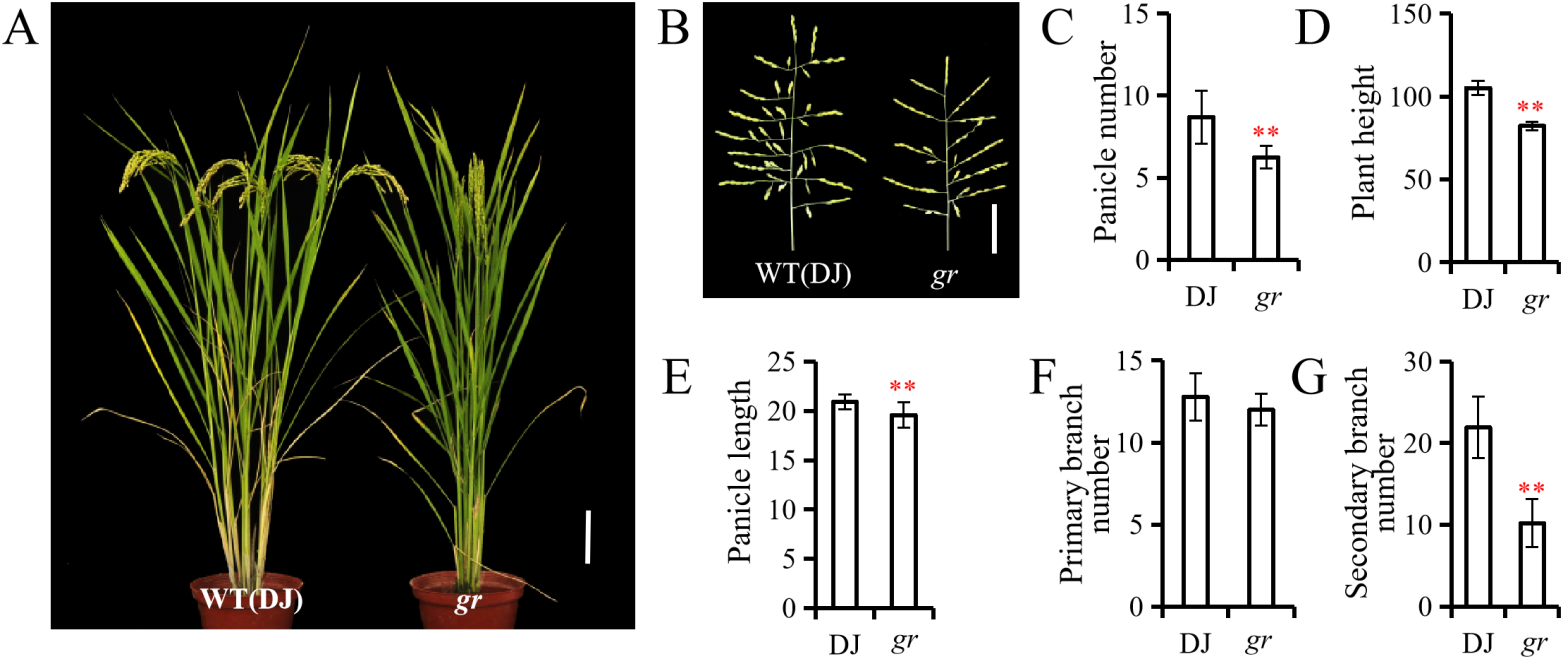
Phenotypic characterization of the *gr* mutant. **(A)** Plant architecture of *gr* and wild type at the heading stage (scale bar = 10 cm). **(B)** Panicle architecture of *gr* and wild type (scale bar = 5 cm). **(C–G)** Phenotypic comparison of panicle number, plant height, panicle length, primary branch number, and secondary branch number between the *gr* mutant and wild type. Data are presented as means ± SE of biological replicates (n = 10). Statistical significance was assessed using Student’s t-test (*P < 0.05, **P < 0.01).

To preliminarily verify the role of *GR* in regulating panicle number, the T-DNA mutant *gr* in the Dongjin background was obtained from the Salk Institute Genomic Analysis Laboratory and genotyped to isolate homozygous lines. Genomic analysis confirmed T-DNA insertion in the first exon of *GR* (**Supplementary Table 9**). Phenotypic evaluation showed clear morphological differences between *gr* and WT (**Figure 6**, **Supplementary Figure 14**). The mutant panicle number decreased from 8.69 to 5.67 compared with WT (34.75%, *P* < 0.01). In addition, plant height, panicle length, and secondary branch number decreased by 19.66%, 5.59%, and 54.44%, respectively (*P* < 0.01). Co-segregation analysis showed that the T-DNA insertion co-segregated with the panicle number phenotype in a segregating population derived from self-fertilization of a heterozygous mutant (*GR*/gr). Allele distribution in LAN and IMP indicated that favorable *GR* alleles have not been widely used in modern breeding. Therefore, naturally occurring favorable *GR* alleles may improve yield and may be valuable for rice breeding.

## 4 Discussion

### 4.1 Utility of natural-population QTLs in germplasm improvement

Based on multi-environment phenotypic evaluations of panicle number in two natural populations (MCC and EMCC) across subtropical (SY), temperate (BJ), and middle (CS) zones to maximize phenotypic variation, we identified various QTLs associated with panicle number through large-scale GWAS. These included 54 environmentally sensitive QTLs in *indica*, 33 environmentally sensitive in *japonica*, 11 environmentally sensitive shared between subpopulations, 9 environmentally stable QTLs in *indica*, and 7 environmentally stable QTLs in *japonica*. Most QTL alleles showed negative effects. Minor-effect QTLs, which are abundantly enriched in IMP varieties, hold considerable value for modern breeding. In contrast, major-effect QTLs remain underutilized, likely due to their association with detrimental traits. This pattern may reflect the “source–sink–flow” balance in rice growth and development (Wang C. et al., 2025). Under limited nutrient conditions, resource trade-offs occur among traits, including negative correlations between panicle number and (i) panicle size, (ii) grain number per panicle, and (iii) grain weight (Wu et al., 2024). These genetic trade-offs are difficult to overcome, limiting the use of major-effect QTLs for panicle number in breeding programs. Furthermore, breeders have favored environmentally stable QTLs with positive effects during rice improvement, whereas environmentally sensitive QTLs have mainly been used to develop LAN varieties based on origin and provenance. Environmentally stable QTLs are preferred for commercial breeding because they consistently influence target traits across locations and seasons (Ye et al., 2025). This stability supports the development of widely adaptable varieties with desirable traits and reduces production risks from environmental fluctuations. In contrast, environmentally sensitive QTLs show strong genotype-by-environment interactions, indicating high adaptability to specific agro-ecological conditions (Masoodi et al., 2025). These QTLs provide a valuable genetic basis for developing rice varieties tailored to diverse environmental challenges.

### 4.2 Efficient screening of functional genes for complex traits

Slow linkage disequilibrium decay, particularly in self-pollinated crops such as rice, often results in large genomic intervals for QTLs identified through GWAS (He and Gai, 2023). In the current study, seven key QTLs for panicle number were fine-mapped using an integrated approach involving haplotype analysis, pathway analysis, Ho-LAMap, and gene annotation. The numbers of candidate genes for *qPN2-1*, *qPN7-1*, *qPN7-2*, *qPN2-2*, *qPN6-1*, *qPN11-1*, and *qPN12–1* were reduced from 323, 1,292, 100, 80, 162, 934, and 146 to 14, 10, 7, 4, 8, 0, and 2, respectively. Haplotype variance analysis is a key method for identifying candidate genes, treating multiple tightly linked variants (e.g., SNPs and InDels) within a QTL region as a single haplotype allele (Nazari-Ghadikolaei et al., 2018). This approach captures linkage disequilibrium more comprehensively, improves detection of candidate causal genes, and increases the precision and strength of association signals. In this study, haplotype variance analysis reduced the total number of candidate genes within the seven QTLs from 3,037 to 1,170. The effects of these genes reflect the cumulative impact of multiple small-effect variants and highlight allelic heterogeneity, where different variants within a gene produce distinct phenotypes (Torra et al., 2021). Pathway analysis of cloned genes and genes within key QTLs was performed using a database developed collaboratively by our laboratory and BGI. Shared pathways, including plant hormone signal transduction, carotenoid biosynthesis, ubiquitin-mediated proteolysis, diterpenoid biosynthesis, amino acid biosynthesis, and carbon metabolism, provide insight into the identification of potential candidate genes (Kanehisa et al., 2022). Among these, hormonal pathways play a central role. Panicle number is shaped by the concerted action of multiple hormonal pathways converging on the underlying genes. *MOC1* are associated with gibberellin (Liao et al., 2019); the histidine phosphotransferases *OsAHP1* and *OsAHP2* function in cytokinin signaling (Sun et al., 2014); *D62* is involved in brassinosteroid biosynthesis (Niu et al., 2022); and the carotenoid cleavage dioxygenases *HTD1* and *D10* participate in strigolactone biosynthesis (Arite et al., 2007; Zhang et al., 2014). Collectively, these findings underscore the integrated, multi-hormonal control of panicle number. Beyond hormonal regulation, additional pathways also contribute to its determination. For instance, Glutamine synthetase *OsGS2* functions in nitrogen metabolism (Cai et al., 2010), supplying substrates for panicle primordium development and protein synthesis, and supporting the energy and material demands of tillering and panicle formation. *MOC2* encodes fructose-1,6-bisphosphatase (Koumoto et al., 2013) and catalyzes the cytosolic conversion of triose phosphates to sucrose in leaves during the day, providing photosynthates and energy that promote tiller-bud growth and panicle organ development. Concurrently, Ho-LAMap, which integrates GWAS and linkage analysis, was used to narrow QTL intervals and directly identify candidate genes for complex agronomic traits (when sufficient sequenced parents were available). In our analysis, we obtained linkage mapping results based on several sequenced biparental populations (*02428 × Nanjing 11, Zhengshan 97B × IRAT109, XQZ B × R9308, and Yuefu × IRAT109) and estimated the heterozygosity index for overlapping genomic regions detected by both GWAS and linkage mapping using a simple statistical model. This approach enabled rapid, precise, and cost-effective delimitation of the minor-effect QTLs *qPN2-1*, *qPN7-1*, and *qPN7–2* associated with panicle number. Fine mapping of *qPN7–1* through Ho-LAMap using polymorphic SNPs from XQZ B × R9308 further reduced the candidate genes from 12 to 10. Additionally, GWAS meta-analysis (Yang L. et al., 2025; Díaz-Muñoz et al., 2026) and colocalization analysis (Yalcinbas et al., 2024; Li et al., 2026) can also be used for fine-mapping QTL to genic resolution. Overall, this integrated strategy effectively narrows QTL intervals, prioritizes candidate genes for functional validation, and is broadly applicable across traits and crop species.

### 4.3 *GR*, *ETR3* and *OsARF25* as valuable genetic resources for improving rice panicle number

Identifying key genes controlling panicle number is vital to breaking the bottleneck of rice yield and to developing “Green Super Rice” by integrating multiple beneficial genes (Yu et al., 2020). This study employed multi-population GWAS, comprehensive fine-mapping strategies and preliminary functional validation to show that the gibberellin receptor *GR* (whose expression pattern closely matches that of the tiller regulator *D10*), the ethylene receptor *ETR3* (from the same family as *ETR2*), and the auxin response factor *OsARF25* (which regulates its downstream target *OsCKX4*) influence cell division, differentiation, and organ development, thereby determining panicle number in rice. From a breeding perspective, genetic variation in *OsARF25* occurs in both indica and japonica accessions, and its favorable allele has been widely used in modern breeding programs. In contrast, elite alleles of *GR* and *ETR3* remain underutilized, indicating strong potential for future genetic improvement. Several genes in hormone pathways also regulate tillering or panicle number, including *SLR1* (Huang et al., 2025) and *APC*/*C^TE^* (Lin et al., 2020) in the gibberellin pathway; *MHZ9* (Huang et al., 2023), *OsEATB* (Qi et al., 2011), and *GNP3* (Ma et al., 2025) in the ethylene pathway; and *OsPIN1* (Xu et al., 2005), *OsPIN2* (Chen et al., 2012), and *SMOS1*/*SMOS2* (Hirano et al., 2017) in the auxin pathway. These genes can be efficiently pyramided via marker-assisted breeding to develop superior cultivars. Although this study provides initial evidence for the roles of *GR*, *ETR3*, and *OsARF25* in regulating panicle number, their upstream - downstream relationships and interaction mechanisms remain unclear. Future studies should focus on their integration within plant hormone regulatory networks and provide deeper mechanistic insight.

## 5 Summary

Here, we performed a genome-wide association study of panicle number using 3.3 million SNPs across 12 phenotypic datasets. We identified 138 QTLs associated with panicle number, most of which showed negative effects, with allele number strongly correlated with the phenotype. Environmentally stable QTLs with small effects appear particularly suitable for breeding. Gene-level analysis further indicated that *OsARF25* and *ETR3* likely play previously unrecognized roles in regulating panicle number. In addition, the gibberellin receptor gene *GR* within *qPN7–2* emerged as a key candidate controlling multiple agronomic traits, including panicle number, plant height, panicle length, and secondary branch number, with an expression pattern similar to *D10*. Collectively, these QTLs and candidate genes from natural populations provide valuable genetic resources for improving rice yield.

## Data Availability

The original contributions presented in the study are included in the article/**Supplementary Material**. Further inquiries can be directed to the corresponding author.
