# Supplementary material for: Identification of QTLs and genes regulating panicle number through genome-wide association studies in Oryza sativa L
Source: Front Plant Sci. 2026 Jun 9;17:1806063. doi: 10.3389/fpls.2026.1806063 (PMC13287107; doi:10.3389/fpls.2026.1806063)
Supplement: Supplementary file 1 [file SupplementaryFile1.docx]

**SUPPLEMENTARY FIGURES**


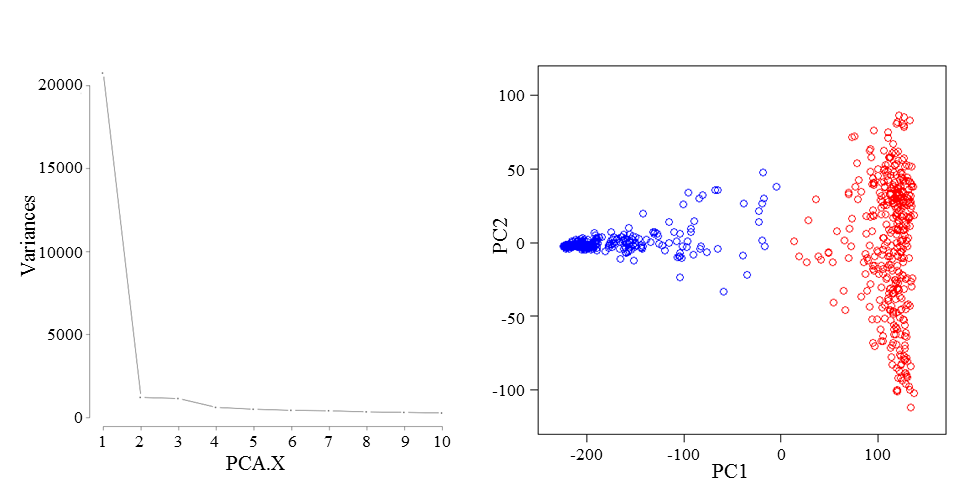


Supplementary Figure 1. Variance distribution of principal components PC1 - PC10 (left) and population structure of 655 varieties shown by a scatter plot of PC1 vs. PC2 (right).

Red dots indicate *indica* varieties; blue dots indicate *japonica* varieties.


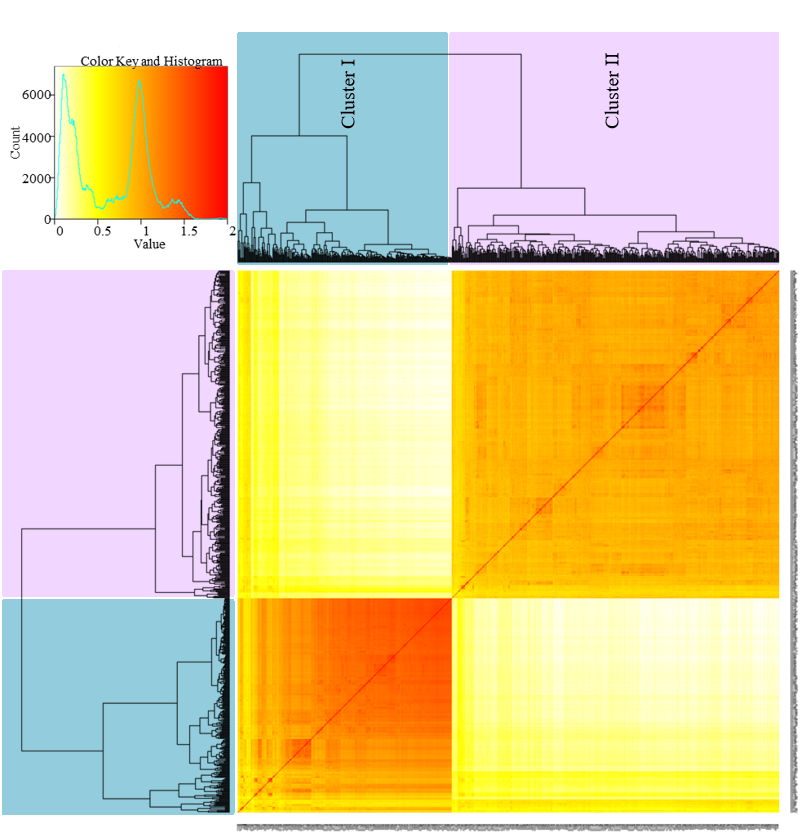


Supplementary Figure 2. Kinship heatmap of 655 varieties.

Cluster I (blue) represents *Oryza sativa* subsp. japonica accessions, and Cluster II (pink-purple) represents *Oryza sativa* subsp. indica accessions.


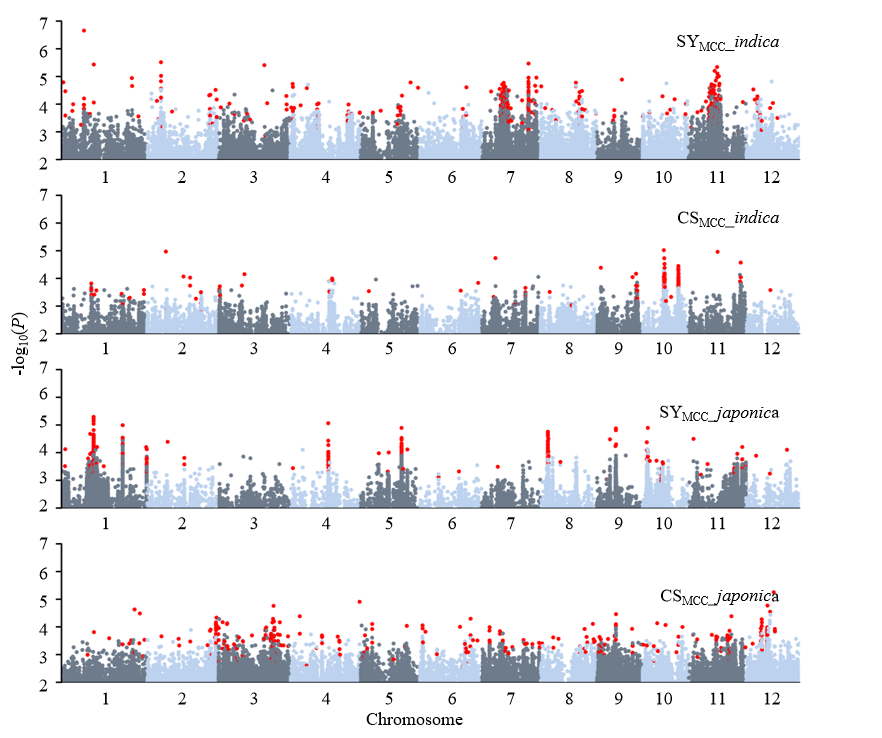


Supplementary Figure 3. Manhattan plots of genome-wide association studies (GWAS) for panicle number in *indica* (*ind*.) and *japonica* (*jap*.) populations in 2013.

Red dots indicate SNPs with -log_10_(*P*) values exceeding the highest -log_10_(*P*) value obtained from 1,000 permutations.


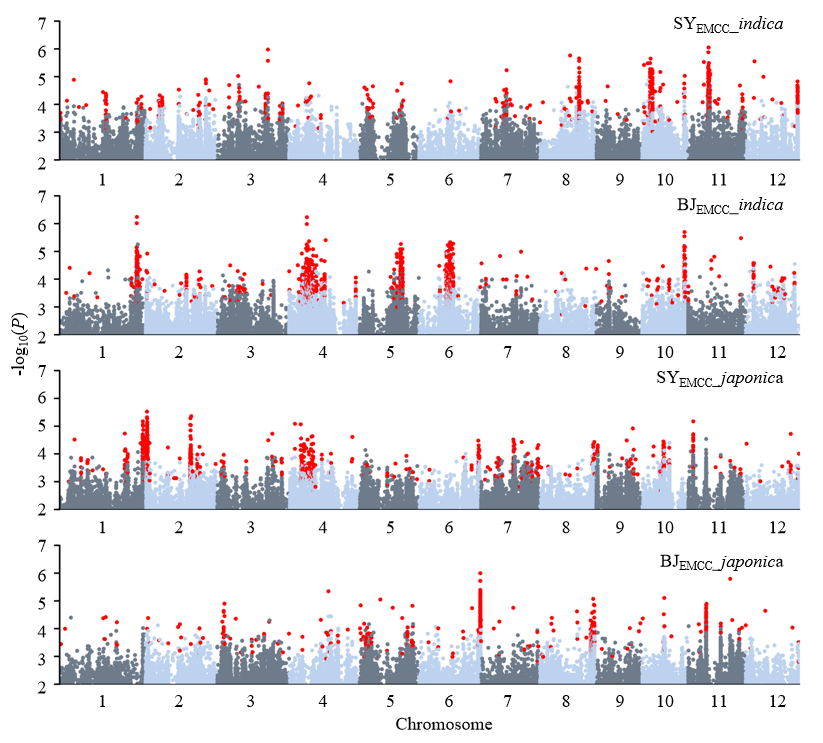


Supplementary Figure 4. Manhattan plots of GWAS for panicle number in *indica* (*ind*.) and *japonica* (*jap*.) populations in 2014.

Red dots indicate SNPs with -log_10_(*P*) values exceeding the highest -log_10_(*P*) value obtained from 1,000 permutations.


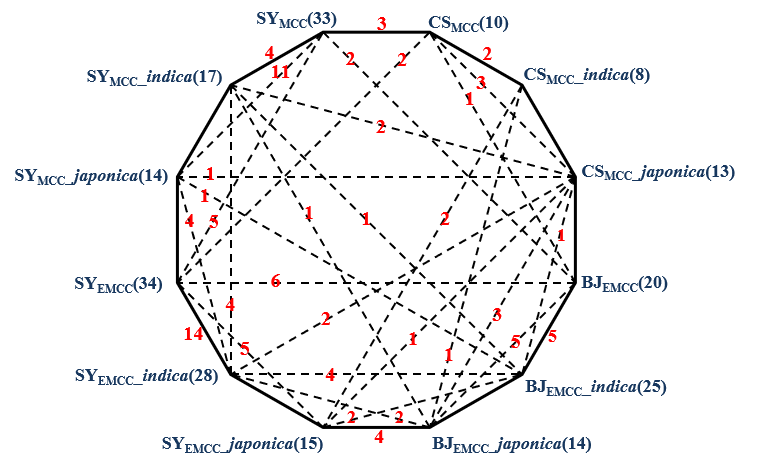


Supplementary Figure 5. Number of **quantitative trait loci (**QTLs) and shared QTLs for panicle number detected by GWAS across different environments and populations.


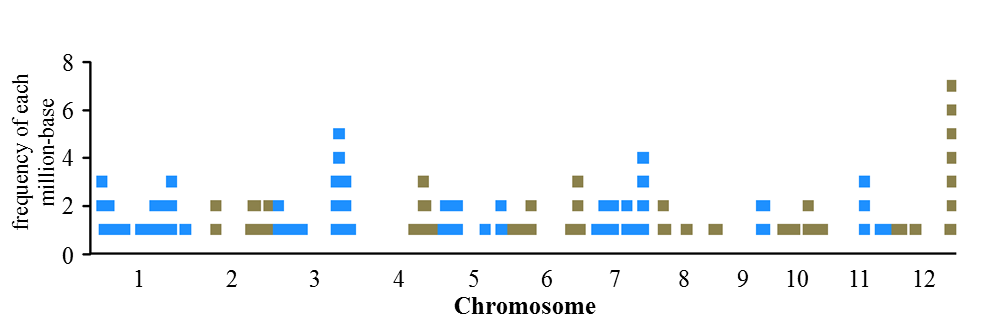


Supplementary Figure 6. Schematic diagram of the frequency distribution of genomic bins containing QTLs identified by linkage analysis for panicle number.


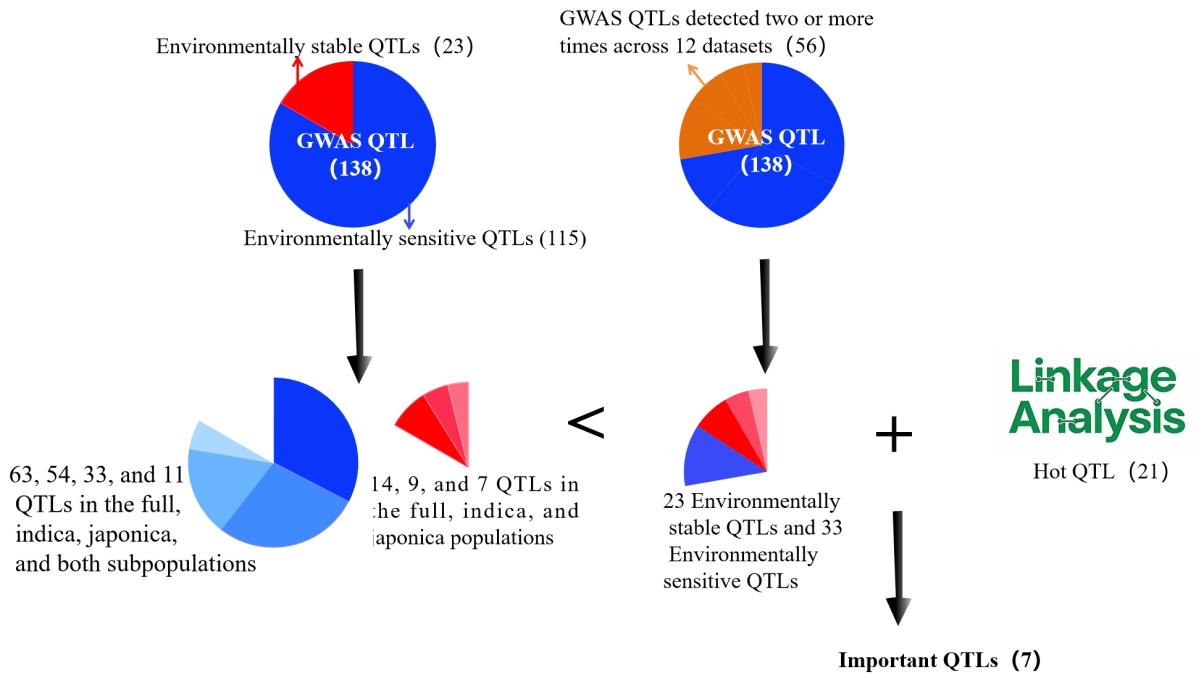


Supplementary Figure 7. Schematic diagram showing the overlap between GWAS QTLs and linkage analysis QTLs.


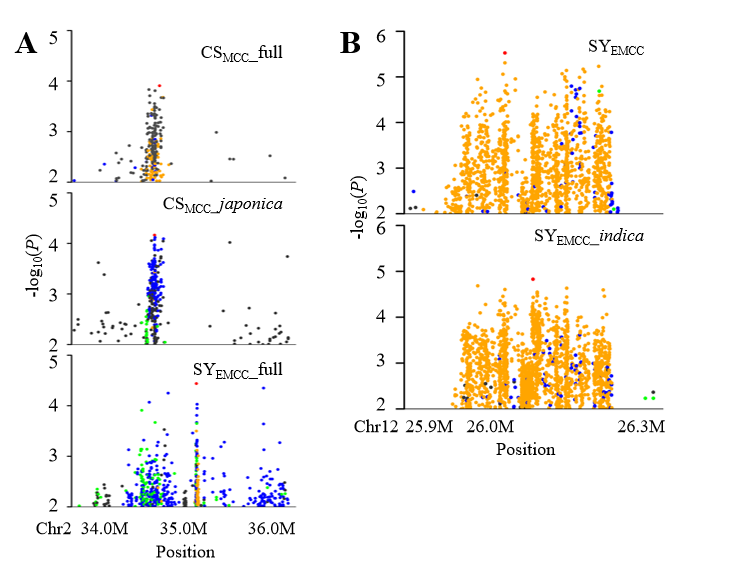


Supplementary Figure 8. Manhattan plots for Chr2:34000000_36000000 (A) and Chr12:25900000_26300000 (B).

LD (*r*^2^) between the lead SNP and all SNPs in the QTLs was calculated. Red, orange, blue, green, and gray dots indicate *r^2^*>0.8, 0.8>*r^2^*>0.6, 0.6>*r^2^*>0.4, 0.4>*r^2^*>0.2, and 0.2>*r^2^*, respectively.


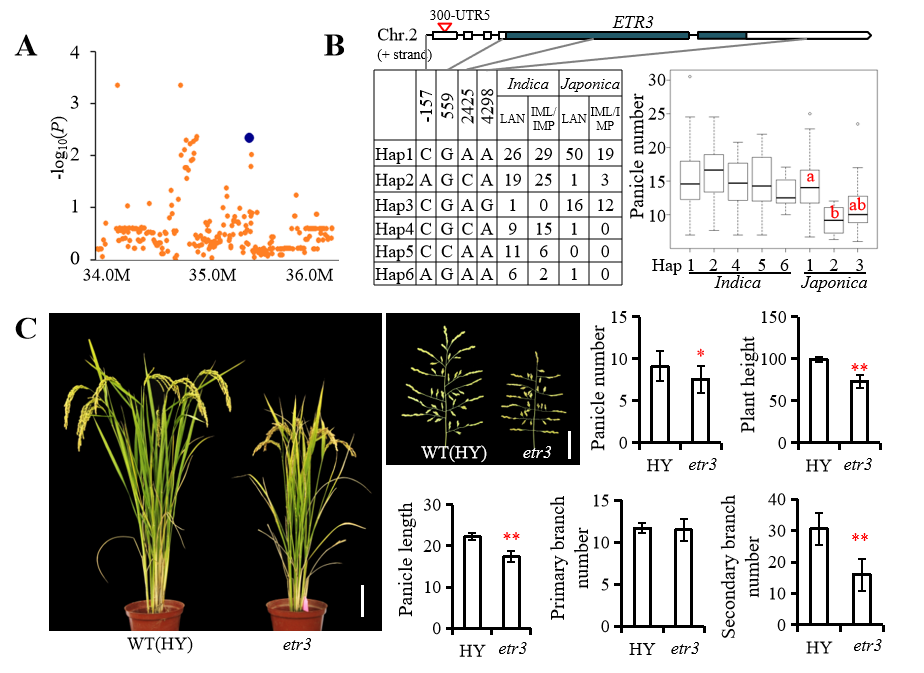


Supplementary Figure 9. Haplotype analysis of *ETR3* and phenotypic characterization of the *etr3* mutant *.*

(A) Haplotype-based variance analysis of candidate genes within *qPN2-2*; the dark blue point indicates *ETR3* (LOC_Os02g57530). (B) Gene structure of *ETR3* and haplotype analysis; the red triangle indicates the T-DNA insertion site. (C) Plant architecture (scale bar = 10 cm), panicle architecture (scale bar = 5 cm) and phenotypic comparison of the *etr3* mutant and wild type*.* Data are presented as means ± standard deviation (n = 10). Statistical significance was assessed using Student’s t-test (**P* < 0.05, ***P* < 0.01).


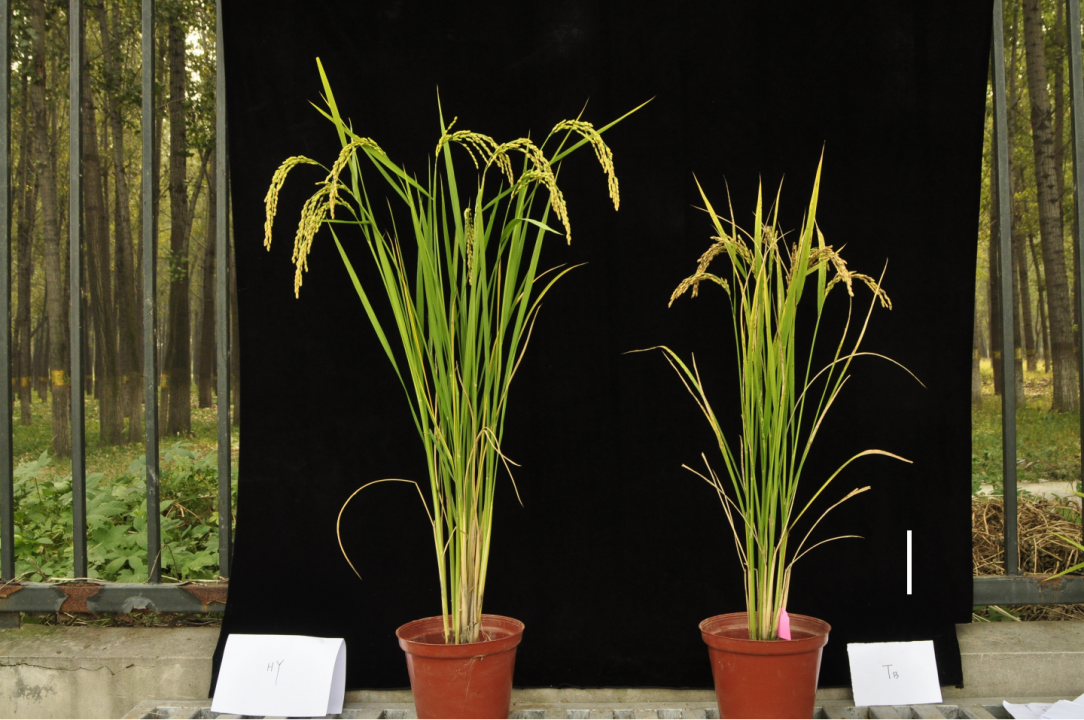


Supplementary Figure 10. Uncropped whole-plant phenotype of wild type and *etr3* mutant plants.

The original, uncropped photograph shows the representative growth phenotype of wild type HY (left) and mutant *etr3* (field code: T13) (right) plants in a single image. Bar = 10 cm.


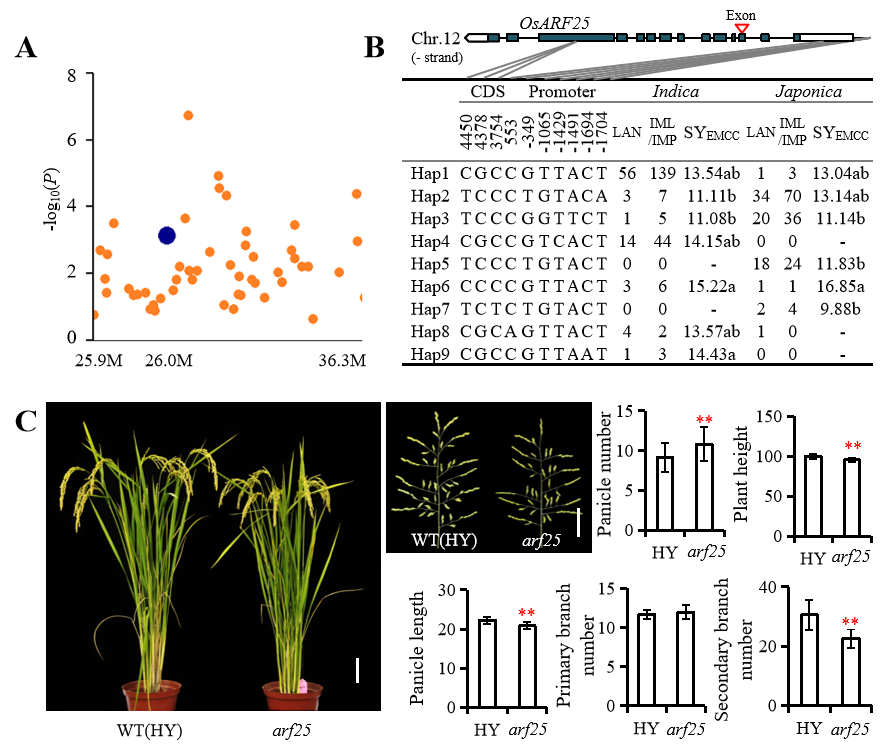


Supplementary Figure 11. Haplotype analysis of *OsARF25* and phenotypic characterization of the *osarf25* mutant.

1. Haplotype-based variance analysis of candidate genes within *qPN12-1*; the dark blue point indicates *OsARF25*. (B) Gene structure of *OsARF25* (LOC_Os12g41950) and haplotype analysis; the red triangle indicates the T-DNA insertion site. (C) Plant architecture (scale bar = 10 cm), panicle architecture (scale bar = 5 cm), and phenotypic comparison of the *osarf25* mutant and wild type. Data are presented as means of biological replicates (n = 10). Statistical significance was assessed using Student’s t-test (**P* < 0.05, ***P* < 0.01).


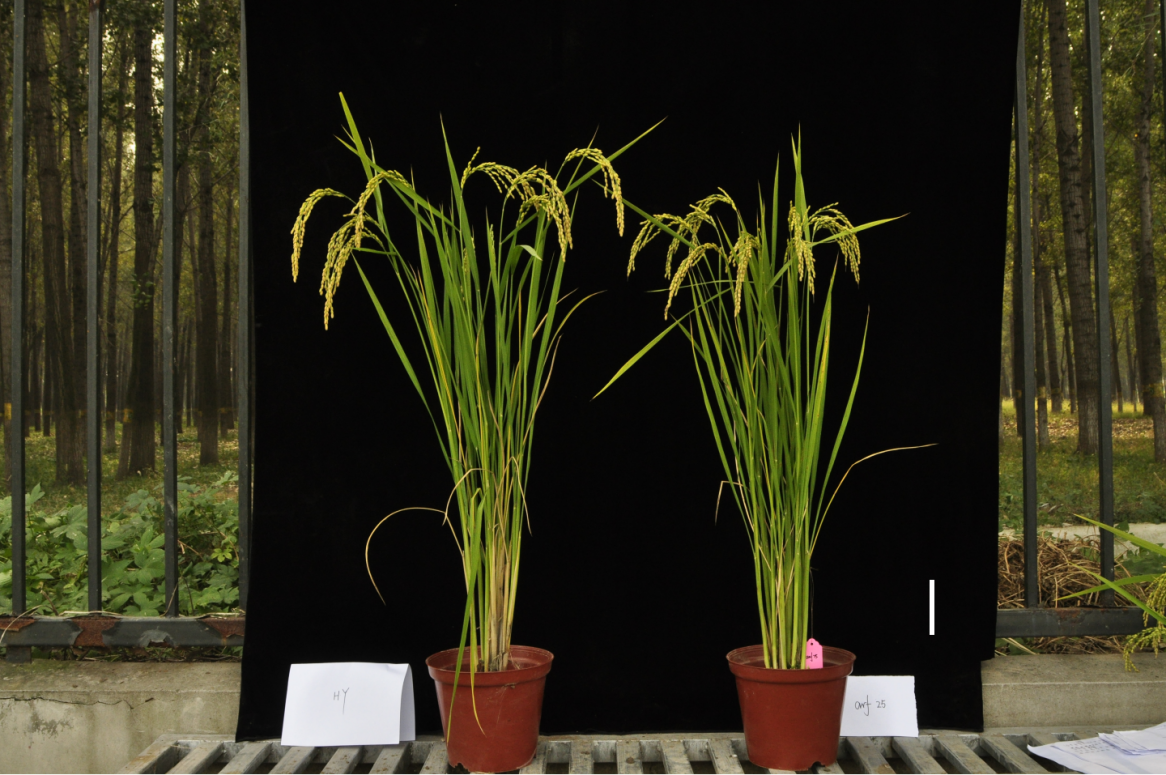


Supplementary Figure 12. Uncropped whole-plant phenotype of wild type and *arf25* mutant plants.

The original, uncropped photograph shows the representative growth phenotype of wild type HY (left) and mutant *arf25* (right) plants in a single image. Bar = 10 cm.


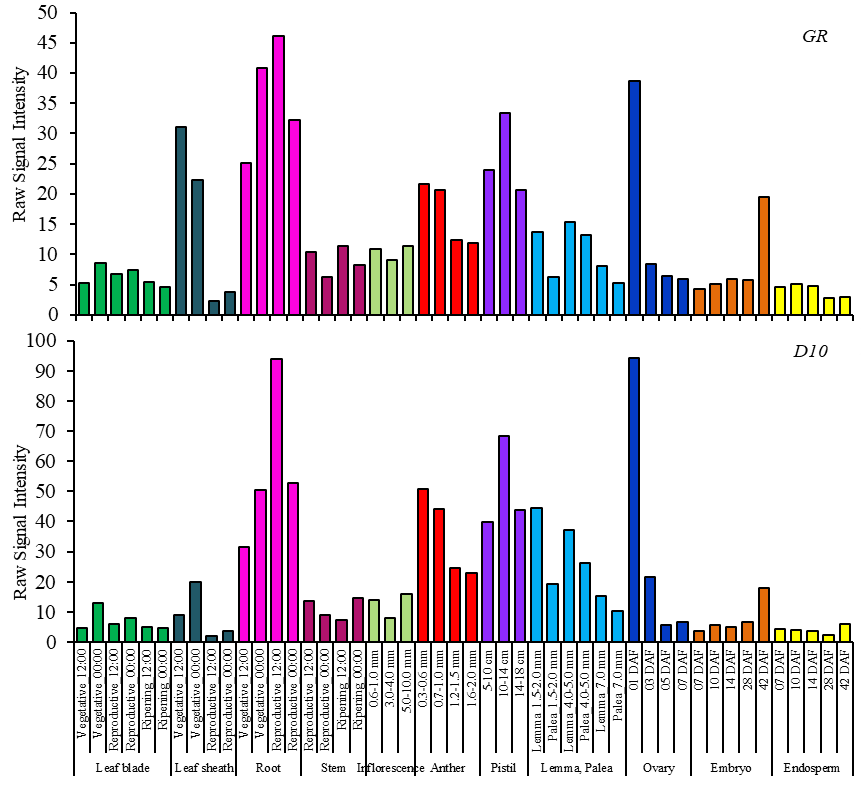


Supplementary Figure 13. Comparison of expression patterns between *GR* and *D10* (http://www.ricexpro.dna.affrc.go.jp/).


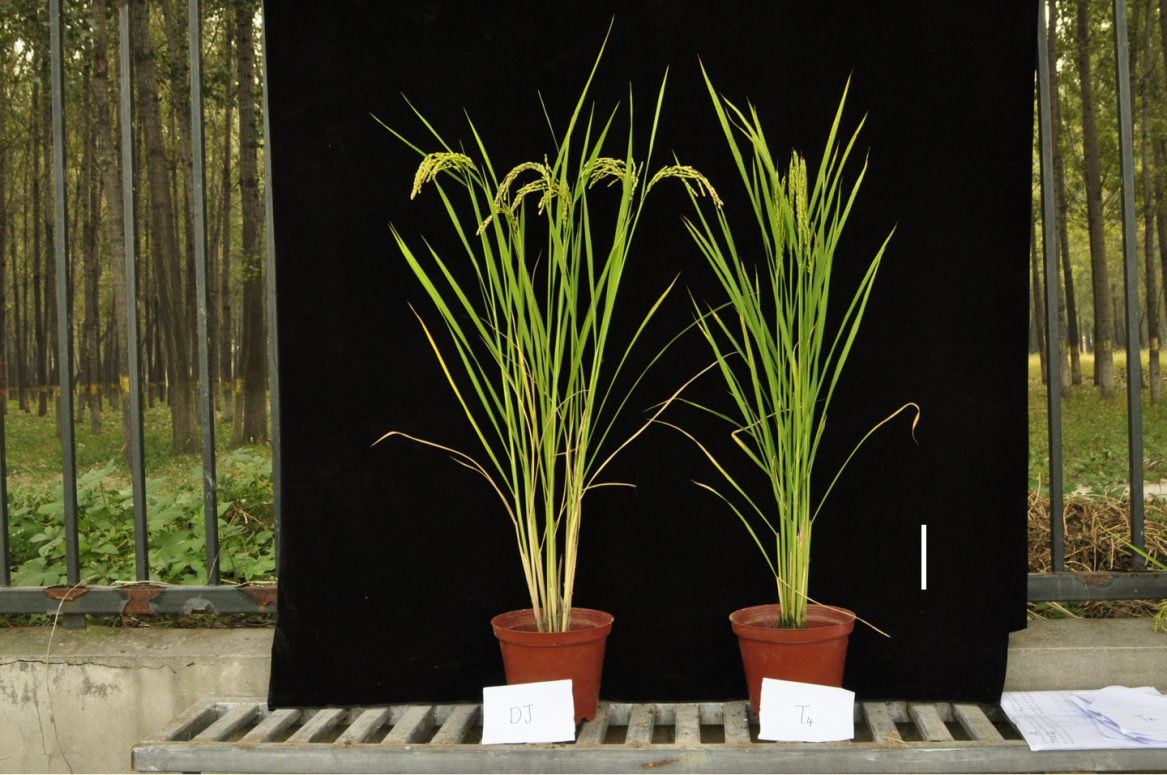


Supplementary Figure 14. Uncropped whole-plant phenotype of wild type and *gr* mutant plants.

The original, uncropped photograph shows the representative growth phenotype of wild type DJ (left) and mutant *gr* (field code: T4) (right) plants in a single image. Bar = 10 cm.
